# The Influence of Exercise Workload Progression Across 36 Sessions of Cardiac Rehabilitation on Functional Capacity

**DOI:** 10.3390/jcdd6030032

**Published:** 2019-09-06

**Authors:** Truman Haeny, Rachael Nelson, Jeremy Ducharme, Micah Zuhl

**Affiliations:** 1 Department of Health, Exercise, and Sports Sciences, University of New Mexico, Albuquerque, NM 87131, USA (T.H.) (J.D.); 2 School of Health Sciences, Central Michigan University, Mt. Pleasant, MI 48859, USA

**Keywords:** exercise, cardiac rehabilitation, cardiovascular diseases, workload

## Abstract

Defining time frames throughout cardiac rehabilitation (CR) to progress exercise workloads may lead to improve functional capacity outcomes. The purpose of this study was to investigate the role of exercise progression on functional capacity among cardiac patients enrolled in CR. This was a retrospective database analysis study. Extracted data included: Demographic, functional capacity (in METs), and exercise intensity during exercise sessions 2, 12, 24, and 36 of CR from 150 patients who completed a 36-session program. Progression of exercise was determined by calculating percent change in treadmill exercise workload within predefined time frames of CR. The time frames were percent change from exercise session 2 to 12 (“%ΔS2–S12), 12 to 24 (%ΔS12–S24), and 24 to 36 (%ΔS24–S36). A multiple linear regression model was developed to predict change in functional capacity (ΔMETs). A significant proportion (21%) of total variation in ΔMETs was predicted by %ΔS2–12, %ΔS12–24, %ΔS24–36, age, sex, and body mass index (BMI). Percent changes between sessions 12 to 24 (%ΔS12–24; β = 0.17, *p* = 0.03) and 24 to 36 (%ΔS24–36; β = 0.23, *p* < 0.01) were significant predictors. Progressing patients between sessions 12 to 24 and 24 to 36 predicted significant changes in functional capacity and reinforced the importance of exercise progression across all 36 sessions of CR.

## 1. Introduction

Cardiac rehabilitation (CR) and secondary prevention programs are effective at improving physical functioning and quality of life among patients with cardiovascular diseases (CVD) and are considered essential for treatment [1,2]. The primary functional outcome among patients following CR is cardiorespiratory fitness, termed functional capacity, which is evaluated according to peak metabolic equivalents (METs). An increase in functional capacity is associated with a reduced risk for clinical events, re-hospitalizations, and all-cause mortality [3,4,5]. For these reasons, exercise programming in CR should be focused on improving exercise capacity. However, a recent survey study among CR facilities found that nearly 60% of clinics reported no defined goals for improving exercise capacity among patients [6].

A possible reason for lack of goal-setting is the large discrepancies in exercise prescription techniques in cardiac rehabilitation [6,7]. Despite a clear understanding that more robust improvements in functional capacity following CR are associated with better health outcomes for cardiac patients, it remains unclear how to most effectively improve functional capacity throughout CR. Various organizations have established clinical guidelines for exercise intensity (%HRmax, RPE), duration (time per session), and frequency (sessions per week), and it appears that an emphasis on achieving higher intensity exercise leads to better outcomes in CR [8]. For example, cardiac patients report greater functional outcomes and have reduced mortality risk when exercised at higher treadmill intensities during 36 sessions of supervised CR [9]. Further, patients who exercise train at less than 3.5 METs after four weeks of CR represent a group at the greatest risk for all-cause mortality [10,11]. The influence of progression in exercise throughout 36 sessions of CR on functional capacity has yet to be examined. In the landscape of cardiac rehabilitation, both session duration and exercise frequency (per week) are traditionally capped due to patient scheduling, facility hours, and staffing. Therefore, exercise intensity is the primary variable that can be manipulated throughout a progressive CR program.

The purpose of this study was to retrospectively investigate the progression of treadmill exercise workload changes within pre-defined stages of cardiac rehabilitation (e.g., exercise sessions 1–12, 12–24, and 24–36) and associated changes in functional capacity among cardiac patients who completed a 36-session CR program.

## 2. Methods

The study was conducted in accordance with the amended Declaration of Helsinki. The University of New Mexico Research Review Board approved the study protocol and waived the need for patient consent due to the retrospective nature of the study. Inclusion criteria were patients who completed a full 36 sessions of cardiac rehabilitation, preformed treadmill exercise during each exercise session, and also completed both baseline and post-rehab submaximal functional capacity testing. The data was extracted between the dates of January 2018 and March 2019.

### 2.1. Cardiac Rehabilitation Program

The outpatient, hospital-based phase II cardiac rehabilitation facility enrolled all patients who qualified for 36 sessions of CR according to the Centers for Medicare and Medicaid Services. Upon intake, patients completed a baseline treadmill test to assess peak functional capacity (peak METs). The staged test began at 1.62 km/h (1 mph) and 0% grade and increased 0.80 km/h (0.50 mph) per minute for the first 4 min. The grade was then increased 2% per minute thereafter. The test was terminated upon the patient reaching 85% of predicted maximal heart rate (using 220—age), or if relative (e.g., shortness of breath, hypertensive response) or absolute (e.g., poor perfusion, moderate-to-severe angina) exercise indications occurred [12]. Among patients taking heart rate suppressing medications (i.e., beta-adrenergic blockers), the termination criterion was a rating of perceived (RPE) exertion of 7 (i.e., hard) using a 1–10 scale. The exercise portion of the CR program took place three days per week for a total duration of 60 min, which included check-in, aerobic exercise training, resistance exercise training, and check-out. Patients were encouraged to complete 36 sessions within 12 weeks (frequency = 3 days/wk) and to attain 150 min per week of aerobic exercise. Therefore, each session was primarily focused on aerobic-based exercise and achieving 50 min per session. The program was multimodal, but only patients who adopted their primary mode as treadmill were included in the study. The reason was to adequately assess exercise workloads during each session. Exercise intensity target for the initial exercise session was 75% of HRmax or an RPE of 5 out of 10 (i.e., moderate intensity). The duration goal for the first exercise session was 16–24 min, with patients completing 4–6 min of exercise using four different modes (treadmill, recumbent machine, cycle ergometer, elliptical). Exercise was progressed every three sessions by increasing workload but keeping the RPE at 5 out of 10 (moderate). Heart rate was monitored each session but was not consistently recorded. Exercise workloads (speed and grade) were documented during each exercise sessions. Upon completion (all 36 sessions), a post-rehabilitation functional capacity test was performed.

### 2.2. Data Extraction

Demographic information was collected, including age, gender, cardiovascular disease type, and body mass index (BMI) classification. Pre- and post-peak functional capacity results were recorded as peak METs. Treadmill exercise workloads in METs were recorded every 12th session. However, session 2 was used as the first exercise session. The reason is that the first CR session was used for orientation, and no exercise was performed. Therefore, exercise workloads in METs was extracted for CR sessions 2, 12, 24, and 36. To assess exercise progression, the percent increases in treadmill exercise METs were calculated from sessions 2 to 12 (“%ΔS2–S12”), 12 to 24 (“%ΔS12–S24”), and 24 to 36 (“%ΔS24–S36”). 

### 2.3. Calculations

The change in functional capacity was calculated as the difference between pre-peak functional capacity (“Pre-METs”) and post-peak functional capacity (“Post-METs”). The MET change was chosen as the outcome variable because of the prognostic value of the measure [13]

METs change = Post-METs − Pre-METs(1)

Exercise intensity was calculated based on the established ACSM formula with S (meters/min) representing treadmill speed and treadmill grade identified as G (% incline) in the equation [14]. The VO_2_ value calculated from speed and grade was converted to METs to standardize exercise workload and used to calculate percent change in exercise intensity (Equation (2)).

METs = (VO_2_ = [0.1 × S] + [1.8 × S × G] + 3.5)/3.5 (2)

Percent changes in exercise intensity between CR exercise sessions 2 to 12, 12 to 24, and 24 to 36 were calculated based on exercise intensities from Equation (2). For example, Final METs was the exercise workload (calculated in METs) for exercise session 12, and Initial METs was the exercise workload (calculated in METs) for session 2. The same calculation was used for exercise sessions 12 to 24 and 24 to 36.

%Δ METs= (Final METs − Initial METs)/Initial METS(3)

### 2.4. Statistics

Descriptive statistics include means, standard deviations, and range for continuous variables, including age, body mass, height, BMI, pre- and post-cardiac rehabilitation functional capacity (pre-METs and post-METs), change in exercise capacity (METs change), and percent changes in treadmill exercise METs from exercise session 2 to 12 (“%Δ2–12”), 12 to 24 (“%ΔS12–24”), and 24 to 36 (“%ΔS24–36”). Frequencies were calculated for categorical variables (i.e., sex and cardiovascular disease history). A multiple regression analysis was performed with METs change as the dependent (outcome) variable. The primary predictors in the model were percent changes in exercise intensity (i.e., %ΔS2–12, %ΔS12–24, and %ΔS24–36). Age, BMI, and sex were also included as confounding variables in the complete model. A hierarchal regression was conducted in which predictors were force entered into each model. Normality of residuals and homoscedasticity were assessed using P-P plot. Multicollinearity was evaluated using the variance inflation factor (VIF) value, along with correlations between predictors. The unstandardized regression coefficients (B) with standard error and standardized β for each predictor were calculated. *p* values < 0.05 were considered significant in the final model. The sample size was determined based on *a priori* analysis using a power of 0.80 and range of effect size (f^2^) from small to large. The calculation was made for five predictors in the model using statistical software (G-Power, University of Dusseldorf, Dusseldorf, Germany). A sample size of 150 patients was selected based on the output.

## 3. Results

### 3.1. Participant Characteristics

Data from a total of 150 (male = 110, female = 40) cardiac rehabilitation patients were included in the analysis. The descriptive and frequency statistics are presented in Table 1. The average percent change in treadmill exercise workload between session 2 and 12 (%ΔS2–S12) was 7.9 ± 7.8%. Between sessions 12 to 24 (%ΔS12–S24), the average percent change in treadmill exercise workload was 4.8 ± 5.6%, and average percent change between sessions 24 to 36 (%ΔS24–S36) was 2.8 ± 4.9%.

### 3.2. Multiple Linear Regression for Functional Capacity Change

The regression models are presented in Table 2. Model four (Table 2, Model 4) suggests that a significant proportion of the total variation in change in METs was predicted by %ΔS2–12, %ΔS12–24, %ΔS24–36, age, sex, and BMI, with the final model accounting for 21% of the variance in functional capacity. A closer inspection of Model 4 shows that the percent changes between sessions 12 to 24 (%ΔS12–24) and sessions 24 to 36 (%ΔS24–36) were significant predictors. According to the unstandardized B, an increase in treadmill intensity by 1% between sessions 12 to 24 predicted a 0.050 increase in METs change. A 1% increase between sessions 24 to 36 predicted an increase of 0.08 METs. Age and the female gender were significant negative predictors. Visual inspection of the P-P plots revealed no violations of normality of residuals and homoscedasticity. We did observe low level collinearity between %ΔS2–12 vs. %ΔS24–36 (r^2^ = 0.153, *p* = 0.031), but the VIF ranged from 1.02–1.10 for all predictors.

## 4. Discussion

The overall objective of this study was to retrospectively investigate progressive changes in treadmill exercise intensity every 12 sessions of a 36-session cardiac rehabilitation program. In a representative sample of patients enrolled in CR, our data suggests that an increase in exercise workload between sessions 12 to 24 and 24 to 36 are the most influential time periods to elicit improvements in functional capacity. A practical interpretation of the data shows that a 10% increase in treadmill exercise workload between sessions 12 to 24 and sessions 24 to 36 predicts a 0.50 and 0.80 MET change in functional capacity, respectively. This reinforces the importance of emphasizing increased progression of exercise across all 36 sessions of CR to improve functional capacity.

Previous research on exercise training workloads in CR has focused on all-cause mortality as the primary outcome [10,11]. Researchers have demonstrated that a 1 MET increase in functional capacity as a result of CR is associated with a 25–40% decrease in risk for all-cause mortality [4,5,15]. These studies used patients who completed an average of 12 CR sessions (minimum = 9; maximum = 15), observing workload improvements of 0.7 and 0.5 METs for men and women, respectively. Results from the current study suggest that patients who only completed 12 sessions had yet to meet the critical time period for improvement. Interestingly, in the current analysis, the largest increase in exercise intensity occurred within the first 12 sessions (~7.9%) but was not a significant predictor of functional capacity change. Conversely, the percent change across sessions 12 to 24 (~4.8%) and 24 to 36 (~2.8%) were smaller, but the impact on functional capacity was greater. An explanation may be that the treadmill workloads at the later sessions (12, 24, and 36) were higher, and therefore, the percent change within each pre-defined stage was smaller. Conversely, this may also suggest that the benefit of 36 sessions of CR is related to a progressive increase in workload throughout the experience.

The progression of exercise is typically not standardized from one CR center to another, so comparison between different healthcare systems should be done with caution [6,16]. Several vague recommendations for exercise progression have been published, and include a gradual increase in RPE from 1 to 5 units beyond 13 (6–20 scale) per session as tolerated [17]; a systematic increase in exercise duration, frequency, and intensity [14]; and an increase in exercise duration prior to an increase in intensity [18]. Moreover, there was no information about exercise progression recommendations among a recent report on exercise prescription practices from 18 countries [16]. Only recently have research efforts been made to explore the importance of exercise workloads within cardiac rehabilitation in attempt to highlight the importance of exercise progression. Keteyian et al. (2018) illustrated that within a heart failure population, each MET increase in exercise workload at the end of CR was associated with reduced mortality and hospitalizations, but progression was only evaluated from pre- to post-CR. Results from the same CR clinic demonstrated that efforts should be made to increase exercise workload within the first 12 sessions [11]. Specifically, patients that were exercising at less than 3.5 METs after four weeks in CR were at a greater risk for all-cause mortality as the result of a CVD event. The researchers emphasized the importance of CR staff to progress patients above this 3.5 MET value. The percent increase in treadmill exercise workloads within CR was used in the current analysis to better guide clinicians on when to increase exercise intensity. Although achieving 3.5 METs by four weeks (session 12) is an important predictor of mortality, it appears that progressing patient treadmill workloads past the 12-session mark is an important predictor of gains in functional capacity. In summary, the progression of exercise workloads in CR has been recently emphasized and was further supported in the current study. Future efforts should focus on exercise progression techniques for cardiac patients enrolled in CR.

The current data also demonstrate that both age and gender in the model were significant negative predictors of functional capacity change in response to 36 sessions of cardiac rehabilitation. More specifically, aging one year predicted a −0.04 change in peak METs. In larger context, aging by 10 years predicted −0.40 change. Improvement in cardiorespiratory fitness has been consistently reported among older patients. However, compared with younger patients, older patients have shown lower improvements in fitness in response to CR [19,20]. Elderly patients are generally more deconditioned, have comorbidities, and are at higher risk for complications compared to younger patients [21]. For these reasons, clinicians may be apprehensive when prescribing progressive aerobic exercise programs for older patients. In addition, the female gender predicted a −0.61 change in peak METs after completion of CR. Outcomes are poorer among women being treated for cardiac disease, and therefore, researchers and clinicians have suggested tailored programs that focus on barriers to rehabilitation, as rates of participation are much lower among women [22,23]. An emphasis on psychosocial interventions along with exercise therapy have been proposed among women to reduce the feelings of burden on their social contact that is commonly reported [22]. These results highlight the importance of developing individual treatment plans for elderly and female patients enrolled in CR.

## 5. Study Limitation

A limitation of the current study and clearly illustrated by Vromen et al. (2019) and O’Neil et al. (2018) is that, although exercise prescription guidelines for cardiac patients have been established, many CR centers vary in their administration of the prescription. The patients included in this analysis were from a single outpatient clinic. The progression of exercise and exercise capacity outcomes of these patients may not be representative of patients enrolled in CR from other facilities. Therefore, a different influential period may exist for a different CR center. Also, exercise workloads were based on treadmill exercise only. Therefore, the interpretation of exercise progression across a multimodal program was limited. Lastly, only sessions 2, 12, 24, and 36 were analyzed, and may not fully represent the exercise workloads completed across the entire 36-session program.

## 6. Conclusions

It is well-established that increasing functional capacity as a result of exercise-based cardiac rehabilitation is linked to improved health outcomes. It is less known how specific variables (mode, frequency, intensity, duration) of the exercise prescription influence functional capacity outcomes. The results of this retrospective study provide insight into the importance of progressing exercise workloads throughout 36 sessions of CR.

## Figures and Tables

**Table 1:** Participant demographics, cardiometabolic health history, anthropometrics, functional capacity before and after cardiac rehabilitation, and exercise intensity during cardiac rehabilitation. N = 150.

Variable	Mean ± SD	Range
Sex (%)		
Male	110	
Female	40	
Cardiometabolic Health History (n)		
CAD	51	
MI	35	
PCI	7	
CABG	8	
Valve replacement (AVR, MVR)	23	
CHF	26	
Age (years)	69 ± 9	38–93
Height (cm)	170.6 ± 8.8	147–191
Body mass (kg)	80.7 ± 17.5	45–139
Body mass index (kg/m^2^)	27.6 ± 4.7	17–41
Pre-METs	4.7 ± 1.9	2.2–10.1
Post-METs	7.0 ± 2.5	2.5–12.9
METs change	2.3 ± 1.7	−0.87–7.2
Exercise Training Intensity		
ΔS2–S12 (%)	7.9 ± 7.8	−12.1–30.25
ΔS12–S24 (%)	4.8 ± 5.6	−22.1–25.5
ΔS24–S36 (%)	2.8 ± 4.9	−15.3–26.9

CAD—coronary artery disease; MI—myocardial infarction; PCI—percutaneous coronary intervention; CABG—coronary artery bypass graft; AVR—aortic valve replacement; MVR—mitral valve replacement; CHF—chronic heart failure; METs—metabolic equivalents; ΔS2-S12—percent change in exercise workload from cardiac rehabilitation (CR) session 2 to 12; ΔS12–S24—percent change in exercise workload from CR session 12 to 24; ΔS24–S36—percent change in exercise workload from CR session 24 to 36.

**Table 2:** Multiple linear regression of change in functional capacity.

	B (SE)	Standardized β	*p* Value
**Model 1**			
Constant	2.06 (0.19)		
%ΔS2–12	0.028 (0.17)	0.13	0.104
R^2^ = 0.01, *p* = 0.10			
**Model 2**			
Constant	1.71 (0.22)		
%ΔS2–12	0.03 (0.01)	0.15	*p* = 0.54
%ΔS12–24	0.06 (0.02)	0.22	*p* = 0.006
R^2^ = 0.06, *p* = 0.006			
**Model 3**			
Constant	1.52 (0.22)		
%ΔS2–12	0.02 (0.01)	0.11	*p* = 0.136
%ΔS12–24	0.06 (0.02)	0.22	*p* = 0.004
%ΔS24–36	0.08 (0.02)	0.25	*p* = 0.002
R^2^ = 0.13, *p* = 0.002			
**Model 4**			
Constant	6.31 (1.46)		
%ΔS2–12	0.01 (0.01)	0.06	*p* = 0.391
%ΔS12–24	0.05 (0.02)	0.17	*p* = 0.03
%ΔS24–36	0.08 (0.02)	0.23	*p* = 0.002
Age	−0.04 (0.01)	−0.24	*p* = 0.003
Gender	−0.61 (0.28)	−0.16	*p* = 0.03
BMI	−0.05 (0.02)	−0.15	*p* = 0.053
R^2^ = 0.21, *p* = 0.003			

%ΔS2–S12—percent change in exercise workload from CR session 2 to 12; %ΔS12–S24—percent change in exercise workload from CR session 12 to 24; %ΔS24–S36—percent change in exercise workload from CR session 24 to 36.
